# Electronegativity determination of individual surface atoms by atomic force microscopy

**DOI:** 10.1038/ncomms15155

**Published:** 2017-04-26

**Authors:** Jo Onoda, Martin Ondráček, Pavel Jelínek, Yoshiaki Sugimoto

**Affiliations:** 1 Department of Advanced Materials Science, Graduate School of Frontier Sciences, University of Tokyo, 5-1-5 Kashiwanoha, Kashiwa, Chiba 277-8561, Japan; 2 Division of Electrical, Electronic and Information Engineering, Graduate School of Engineering, Osaka University, 2-1 Yamada-Oka, Suita, Osaka 565-0871, Japan; 3 Department of Thin Films and Nanostructures, Institute of Physics, Czech Academy of Sciences, Cukrovarnická 10/112, Prague 16200, Czech Republic; 4 Regional Centre of Advanced Technologies and Materials, Department of Physical Chemistry, Palacky University, Šlechtitelů 27, 78371 Olomouc, Czech Republic

## Abstract

Electronegativity is a fundamental concept in chemistry. Despite its importance, the experimental determination has been limited only to ensemble-averaged techniques. Here, we report a methodology to evaluate the electronegativity of individual surface atoms by atomic force microscopy. By measuring bond energies on the surface atoms using different tips, we find characteristic linear relations between the bond energies of different chemical species. We show that the linear relation can be rationalized by Pauling's equation for polar covalent bonds. This opens the possibility to characterize the electronegativity of individual surface atoms. Moreover, we demonstrate that the method is sensitive to variation of the electronegativity of given atomic species on a surface due to different chemical environments. Our findings open up ways of analysing surface chemical reactivity at the atomic scale.

## Abstract (web summary)

Electronegativity is a fundamental concept in chemistry; however it is an elusive quantity to evaluate experimentally. Here, the authors estimate the Pauling electronegativity of individual atoms on a surface via atomic force microscopy using a variety of chemically reactive tips.

Electronegativity, an important theoretical concept in chemistry, was originally defined by Linus Pauling as ‘the power of an atom in a molecule to attract electrons to itself'^1^,^2^. Since that time, various classical scales of electronegativity have been suggested^3^,^4^,^5^,^6^, including the rigorous modern formalism of absolute electronegativity developed along with density functional theory (DFT)^7^. In contrast, conventional experimental means for measuring electronegativity have been limited to thermochemical techniques, that is, the measurement of ensemble-averaged bond energies^8^. On the other hand, atomic force microscopy (AFM) was able to achieve atomic resolution on both semiconductor^9^ and insulator^10^ surfaces. It has also found numerous applications in the field of chemistry: for example, quantitative measurement of short-range chemical forces^11^, chemical identification of individual surface atoms^12^, visualization of the internal structures of organic molecules^13^,^14^,^15^,^16^ and metal clusters^17^, discrimination of the Pauling bond order^18^,^19^ and tracking of surface chemical reactions^20^,^21^.

Here, we extend this already impressive suite of applications by demonstrating the use of AFM in characterizing the electronegativity of individual surface atoms. Pauling's electronegativity values for individual single atoms on surfaces can be estimated using site-specific energy spectroscopy acquired with a variety of AFM tips. Namely, we show that the binding energies for individual surface atoms observed using different tips can provide an ensemble of data that can be used later to determine the electronegativity of different chemical elements or chemical groups on a surface (Fig. 1a). Our experimental findings are supported by theoretical analysis based on DFT calculations.

## Results

### Scatter plots of the bond energies

**Figure 1:**
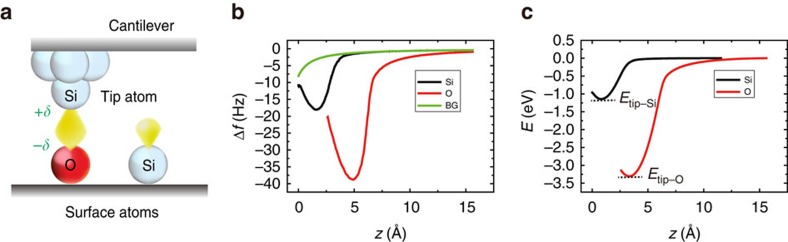
Site-specific energy spectroscopy. (**a**) Schematic illustration of AFM energy spectroscopy with the polar covalent bond of Si–O. (**b**) Δ*f* curves measured on Si and O adatoms on the Si(111)-(7 × 7) surface. The background (BG) Δ*f*(*z*) curve is also included for subtraction of the long-range component (Methods). (**c**) *E*(*z*) curves converted from short-range components in the Δ*f*(*z*) curves in **b**. Bond energies are defined at the minima of *E*(*z*) curves. The acquisition parameters are *f*_0_=152.913 kHz, the oscillation amplitude (*A*)=146 Å, the spring constant (*k*)=28.2 N m^−1^ and sample bias (*V*_S_)=+40 mV.

**Figure 2:**
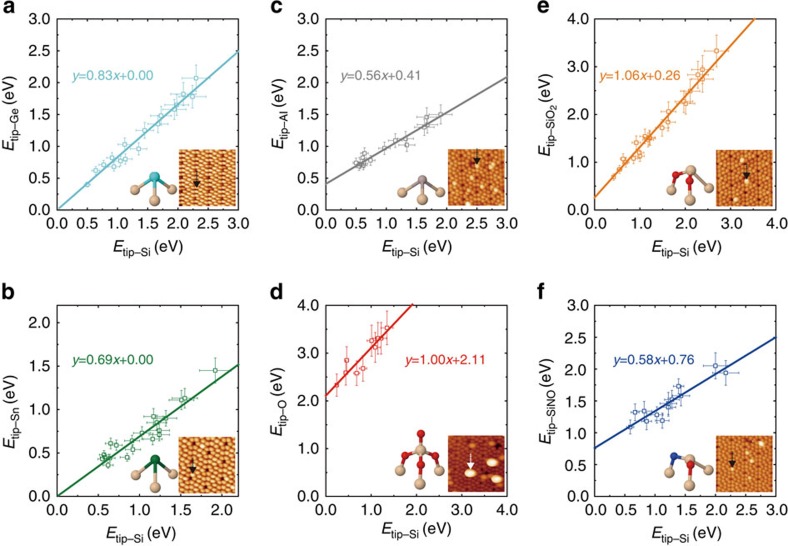
Linear relations between bond energies of different chemical species. Scatter plots of the bond energies of Ge (**a**), Sn (**b**), Al (**c**), O (**d**) adatoms, SiO_2_ (**e**) and SiNO (**f**) complexes obtained experimentally. The bond energies were measured above the bright spots in AFM images as shown in the insets. The individual error bars are estimated as 10% of the corresponding bond energies based on uncertainties in measurements of *A* and *k*. The scatter plots were fitted using weighted orthogonal distance regression.

We demonstrate the concept using a prototypical Si(111)-(7 × 7) surface with different local impurities (Supplementary Figs 1–5; Supplementary Methods 1). To characterize the Pauling electronegativity, we acquired bond energies by detecting the frequency shift (Δ*f*) from the resonant frequency (*f*_0_) of an oscillated Si cantilever at room temperature (Methods). As an example, we show tip-surface distance dependences of Δ*f* (Δ*f*(*z*) curves) measured on Si and O adatoms on the Si(111)-(7 × 7) surface in Fig. 1b. As seen in the inset of Fig. 2d, locally formed silicon oxide has a tetrahedral SiO_4_ structure, which is typical for the basic unit of silicate and stable even at room temperature^22^. By converting the Δ*f*(*z*) curves to a short-range energy-distance curves (*E*(*z*); see Methods), we were able to obtain minimum potential energies (bond energies) on Si 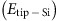 and O 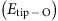 as shown in Fig. 1c. Note that 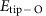 represents one of the strongest single polar covalent bonds ∼3 eV. Once the tip apex was deliberately changed by a tip-modification method, for example, mild contact with the sample surface, the magnitudes of 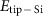 and 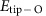 were altered. This tip preparation method ensures that the tip apex would most likely be made of Si. Next, we investigated various sets of 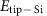 and 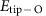 by changing the tip state and then made a scatter plot of the bond energies as shown in Fig. 2d. Similarly, we investigated scatter plots of the bond energies associated with Ge, Sn and Al adatoms on the Si(111)-(7 × 7) surface (Fig. 2a–c). Remarkably, all the scatter plots showed linear relations. Fitting with a straight line, we were able to extract the slopes and intercepts as summarized in Tables 1 and 2.

### Interpretation of the linear relations by Pauling's equation

We rationalized the linear relation between the bond energy of tip apexes and different surface atoms by employing Pauling's equation for the bond energy *E*_A–B_ of heterogeneous polar bonds between atoms A and B^1^,^2^:


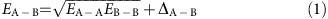


where *E*_A–A_ (*E*_B–B_) represents the energy of homogeneous bond A–A (B–B); 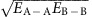 and Δ_A–B_ are covalent and ionic energy parts of *E*_A–B_, respectively. Thus, based on heterogeneous bond energies between the tip-termination atom and target X 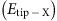 and reference Si 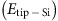 atoms on the surface, which were experimentally obtained using the energy spectroscopy as described above, we deduced the following equation (Supplementary Note 1):


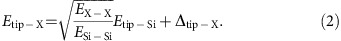


The homogeneous bond energy *E*_X–X_ 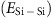 will be discussed next using theoretical models. According to the Pauling theory, the term Δ_tip−X_ represents the ionic energy stemming from the difference in electronegativity between the tip and the target atom X. In this context, the linear dependence observed in Fig. 2 can be interpreted as an experimental confirmation of the validity of Pauling's equation (1) for the electronegativity scale.

### Chemical identification by the slopes

Table 1 shows experimentally determined slopes in the scatter plots of the bond energies. Interestingly, the slope for the Ge and Sn adatoms match the interaction ratios of the maximum attractive forces of the same atomic species determined previously^12^,^23^,^24^. As discussed in ref. 12, the interaction ratio of the maximal forces (or similarly the minimum energies) represents the ‘fingerprint' of the chemical identities. If there is no electronegativity difference between the tip and the target atom X, the term Δ_tip−X_ in equation (2) vanishes and the following relation holds: 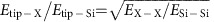. To verify this relation, we calculated the homogeneous bond energies *E*_X−X_ and the related theoretical slopes (Table 1) for various elements (including *E*_Si−Si_) by modelling the interactions between simple mirror-geometry clusters (Supplementary Fig. 6; Supplementary Methods 2). Notably, all calculated values reproduced very well the experimental slopes. Thus, the values of slopes in the scatter plots characterize the covalent bond energy of target X adatoms with tip renormalized by the *E*_Si−Si_ bonding energy. We should stress that the previous chemical identification method^12^ holds only for elements having very similar electronegativity. By contrast, the present identification method based on scatter plots of the bond energies is applicable to arbitrary elements with divergent electronegativity.

### Electronegativity determination by the intercepts

Equation (2) indicates that the intercepts in the scatter plots of the bond energies can be interpreted as the ionic energies, which are correlated with electronegativity values of the tip 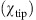 and the X 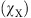 atoms through the equation of 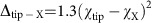 (refs 1, 2). Since atomically sharp tips were prepared by intentional poking into clean regions of the Si surfaces, we assume that their apexes are terminated with Si atoms such that individual 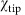 are almost the same magnitude as that of an Si adatom 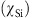 on the Si(111)-(7 × 7) surface. This assumption is justified by the experimental facts that all the scatter plots in Fig. 2 demonstrate the linear relation (Supplementary Figs 7 and 8; Supplementary Note 2), and by theoretical calculations, in which Si–tip models mimicking a surface Si adatom can provide good agreement with experimental evidences^25^. We find the total-energy DFT calculations simulating few Si–tip models (Supplementary Fig. 9) interacting with the Si(111)-7 × 7 slab surface can reproduce well bond energies regarding with Ge, Al and SiO_2_ (discussed later) acquired with tips having large 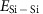 bonding energy >1.0 eV (Supplementary Fig. 10). However, slopes and intercepts estimated only with the theoretical plots do not reproduce exactly the experimental values. We attributed this discrepancy to statistical uncertainty due to very small number of calculated points corresponding to only five highly reactive Si–tip models (for more details, see Supplementary Note 3).

We should note, from the equation of 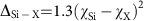, it is evident that only difference between electronegativities of the reference Si tip 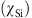 and target X 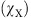 can be directly determined within the experimental precision. Thus, we need calibration of the reference electronegativity 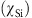 for a quantitative comparison of obtained electronegativity values with those tabulated in the periodic table. Throughout this report, we deliberately assume 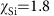 in accordance with the definition in Pauling's electronegativity scale^1^,^2^.

To determine the electronegativity values of individual target X adatoms from the relation 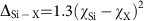, we have to know the sign of the electronegativity differences (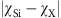 in Table 2). This information is not *a priori* accessible from the present experimental approach. One possibility is to stick with a ‘chemical intuition', when the chemical origin of the impurities is known (for example, Al is less electronegative than Si). Another one is ‘classical' approach to compare a scatter plot obtained by Si tips with another plot acquired by chemically different (such as Al-terminated) tips whose electronegativity is diverged from 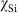. We expect that future work with at least two chemically different tips would give us a clue for the sign of electronegativity differences. For an alternative approach, we consider the modern definition of electronegativity^26^, where the electronegativity is directly related to the chemical potential *μ* (that is, work function): 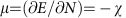, where *E* and *N* represent the total electronic energy and number of electrons, respectively. In the case of surfaces, the electronegativity *χ* can be replaced with a local variation of the work function on a given surface site. The variation of the work function on surfaces with atomic-scale resolution can be estimated by Kelvin probe force microscopy (KPFM)^27^. Unfortunately, the presence of artefacts at close tip–sample distances hampers the reliable interpretation of a KPFM signal at the minimum potential energy^28^. Thus, here, we opted for a theoretical evaluation of the local work functions analysing the Hartree potential (*V*_H_(*z*)) obtained from DFT calculations. We believe, however, that the advancement of KPFM should allow us to determine the sign of the electronegativity difference exclusively by experimental measurements in the near future.

**Figure 3:**
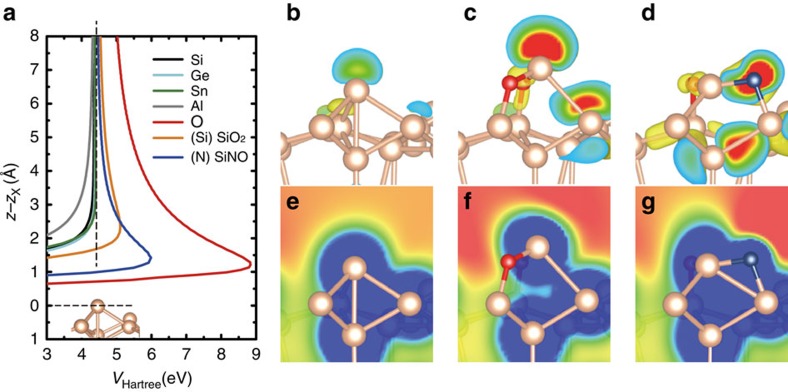
Evaluation of electronegativity difference by DFT calculations. (**a**) A one-dimensional plot showing the variation of the Hartree potential above different adatoms such as Si, Ge, Al, O or N. The zero point of the Hartree potential is set to the Fermi level, and the *z*-distance is aligned to the height of an inspected adatom (*z*_X_) on the Si(111)-(7 × 7) surface. The calculated electron density isosurfaces (0.02 e Å^−3^ in yellow) of the highest occupied frontiers orbital on the Si adatom (**b**), SiO_2_ (**c**) around the Fermi level and SiNO (**d**) located 1 eV below the Fermi level. Cut-plane of the electron density isosurface is coloured from red (0.13 e Å^−3^) to blue (0.02 e Å^−3^). Plotted two-dimensional plane of the Hartree potential projected onto the Si adatom (**e**), SiO_2_ (**f**) and SiNO (**g**) complex (range from 0 (blue) to 5 (red) eV).

Total-energy DFT simulations reveal that the adatoms of various elements on a Si(111)-(7 × 7) surface show variations of local density of states (Supplementary Fig. 11) and electron density (Supplementary Fig. 12) near the Fermi energy, and of *V*_H_(*z*) above the inspected adatom (Supplementary Fig. 13). Figure 3a displays the calculated one-dimensional profiles of *V*_H_(*z*) along the *z* axis, representing the local work functions above the Si, Ge, Sn, Al and O adatoms. Interestingly, the local work functions at a *z*-distance of 3–4 Å above Ge and Sn adatoms are very similar to that of a Si adatom. This observation together with the lack of an intercept in the experimental scatter plots of Ge and Sn (Fig. 2a,b) apparently confirms the above assumption that 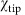 is close to 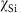; that is, 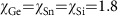 (Table 2). On the other hand, the local work functions of Al and O show effectively lower and higher values, respectively, than the Si adatom shown in Fig. 3a. This means 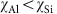 and 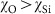, and the electronegativities of Al and O become 1.24±0.04 and 3.07±0.07, respectively. These values are in very good agreement with Pauling's electronegativities (Table 2).

### Evaluation of group electronegativity

In general, the electronegativity of surface atoms (here, Si adatoms) can be modified by their surrounding environments: for example, structural and chemical rearrangement of local structures, charge transfer from the neighbouring atoms and re-hybridization of orbitals. Previously, this information has not been experimentally accessible. From this perspective, the present method provides a unique opportunity to obtain electronegativity values for specific surface atoms in different local chemical structures. To demonstrate such sensitivity towards group electronegativity, we carried out measurements on a ‘SiO_2_' structure (Fig. 2e), where two O atoms are inserted into the back bonds of an Si adatom^22^, and on a ‘SiNO' structure (Fig. 2f) created from dissociated O and N atoms (Supplementary Methods 1). Local atomic arrangements obtained from the total-energy DFT calculations of the SiO_2_ and SiNO structures are presented in Fig. 3c,d, respectively.

Although the topmost atom of the SiO_2_ configuration is Si, the scatter plot of bond energies for SiO_2_ (Fig. 2e) clearly shows an intercept of 0.26±0.08 eV. To understand the presence of the intercept, we analysed the variation of *V*_H_(*z*) for SiO_2_. Figure 3a,e,f shows that the local work function above the Si adatom in the SiO_2_ configuration is higher than that for the pure Si adatom, that is, 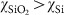. Consequently, we can estimate the electronegativity of the Si adatom in SiO_2_ to be 2.25±0.07, and thus more electronegative than that of pure Si (Table 2). It is worth noting that while the intercept appears, the slope remains close to 1. Thus, it is evident that the local charge transfer in the SiO_2_ structure between the Si adatom and adjacent O atoms changes the ionic energy term Δ but leaves the covalent bond energy almost unaffected. This effect makes sense given that the dangling bond on the Si adatom of SiO_2_ maintains an *sp*_3_ hybridization very similar to that of an Si adatom on a clean surface (Fig. 3b,c), but with a different charge density.

The case of SiNO is more complex. Experimentally, the scatter plot of bond energies for SiNO (Fig. 2f) shows an intercept of 0.76±0.12 eV. The magnitude of the experimental slope is far from unity, as is the theoretical slope for SiNO (Table 1). This reveals that the atomic and electronic structure of the SiNO complex differs from that of a bare Si adatom and/or that the neighbouring N atom as well as the Si atom is involved in the chemical interaction with the Si atom at the tip apex. Indeed, according to the DFT calculations the atomic structure of the SiNO complex is different from the Si adatom, as shown in Fig. 3b,d. The highest occupied frontier orbital of the SiNO structure is delocalized between the Si, N and O atoms (Fig. 3d). Therefore, one should consider the whole structure instead of individual atoms in the electronegativity analysis. To determine the sign of 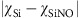, we analysed the spatial distribution of *V*_H_(*z*) over the SiNO structure. Figure 3g shows the result: the work function is maximal at the N atom of SiNO (Fig. 3a), and monotonically decreases towards the Si atom but still remains slightly higher at the relevant *z*-distance than it is over the pure Si adatom; hence, 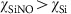. Accordingly, we determined the experimental electronegativity of the SiNO structure to be 2.57±0.06. Compared to the conventional Pauling's electronegativities of Si(1.8) and N(3.0), the obtained electronegativity of the SiNO structure reflects its intermediate character. In sum, the analysis of the SiNO and SiO_2_ complexes indicates that AFM is able to determine the group electronegativity.

In conclusion, we have presented here a method that, for the first time to our knowledge, allows experimental characterization of the electronegativity of individual surface atoms by means of AFM. We have found that the scatter plots of the bond energies can be interpreted based on Pauling's equation for polar covalent bonds. This allowed us to disentangle the covalent and ionic bond energies in polar covalent bonds, and hence to estimate the electronegativity of the individual elements. In addition, this method facilitates analysis of the group electronegativity of atoms belonging to locally formed nanostructures. We expect that the present method can be applied to other interesting systems such as transition metal oxides used in catalysis by terminating AFM tips with known atoms on the surface. Furthermore, we believe that the method can not only characterize the specific electronegativity of individual atoms, defects, adsorbates and dopant impurities on surfaces but may also open up new ways of analysing surface chemical reactivity in terms of surface electrophilicity/nucleophilicity against reactants^29^, and surface chemical softness/hardness with regard to the hard–soft acid–base principle^26^,^30^.

## Methods

### Atomic force microscopy

All experiments were carried out using a custom-built ultrahigh-vacuum AFM system at room temperature with a typical base pressure of 5 × 10^−9^ Pa. An optical interferometer was equipped to detect the cantilever deflection. Frequency-modulation technique was used for obtaining Δ*f*. We used commercial Si cantilevers, whose tip apexes were cleaned by Ar ion sputtering to remove the native oxidation layers. The cantilever was oscillated with sufficiently large amplitude (*A*∼100 Å) for stable AFM operation. We chose the Si(111)-(7 × 7) surface as playground for the purpose of the present experiments since the surface had many Si adatoms on which atoms at tip apex were replaced by mild contact. To compensate a contact potential difference between tip and sample, we properly applied *V*_S_ to the sample while keeping the tip bias at ground.

### Site-specific energy spectroscopy

When we performed site-specific energy spectroscopy, thermal drift was compensated by feed-forward technique^31^ and the precision of lateral position of the point spectroscopy became better than ±0.1 Å, both of which were realized by our atom-tracking implementation^32^. After recording several Δ*f*(*z*) curves on an adatom of interest, we averaged them to a single Δ*f*(*z*) curve for converting to *E*(*z*). Note that energy dissipation during the spectroscopy was negligible. For eliciting accurate bond energies exerted on the foremost tip atom and surface adatoms, we subtracted the background Δ*f*(*z*) curve (Fig. 1b; Supplementary Methods 1), which is obtained by analytically fitting a Δ*f*(*z*) curve measured on a corner hole site^11^,^12^, from Δ*f*(*z*) curves on the individual target and reference adatom sites. Finally, the Δ*f*(*z*) curves only containing the short-range contribution were converted to *E*(*z*) using the inversion procedure suggested by Sader and Jarvis^33^.

### Density functional theory calculations

All DFT calculations have been done using the Vienna *Ab-Initio* Simulation Package (VASP 4.6)^34^,^35^, which is a pseudopotential plane-wave code. The basic versions of ultrasoft Vanderbilt pseudopotentials^36^,^37^ supplied with VASP were used for all chemical elements involved (Si, H, O, N, Al and Ge) apart from Sn, for which the Sn*d* potential that includes *d*-electrons as valence electrons was adopted. The PW91 implementation of the generalized gradient approximation^38^ was chosen to describe the exchange-correlation functional. The size of the plane-wave basis was determined by the cutoff energy of 300 or 396 eV. Only the central (Gamma) point of the first Brillouin zone was considered for the Bloch-wave solutions.

### Data availability

The data that support the findings of this study are available from the corresponding author on reasonable request.

## Additional information

**How to cite this article:** Onoda, J. *et al*. Electronegativity determination of individual surface atoms by atomic force microscopy. *Nat. Commun.* **8,** 15155 doi: 10.1038/ncomms15155 (2017).

**Publisher's note**: Springer Nature remains neutral with regard to jurisdictional claims in published maps and institutional affiliations.

## Supplementary Material

Supplementary Information Supplementary Figures, Supplementary Notes, Supplementary Methods and Supplementary References

## Figures and Tables

**Table 1:** Summary of experimental and calculated slopes 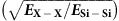 .

**Bond configuration**	**Slope (exp.)**	**Slope (cal.)**	***E***_**X−X**_ **by DFT (eV)**
Si–Si	1	1	2.20
Ge–Ge	0.83±0.07	0.83	1.52
Sn–Sn	0.69±0.07	0.76	1.27
Al–Al	0.56±0.07	0.46	0.47
O–O	1.00±0.26	0.89*	–
O_2_Si–SiO_2_	1.06±0.07	1.17	2.99
ONSi–SiNO	0.58±0.11	0.41	0.37

^*^We referred to single bond energies in ref. 2: Si–Si, 1.83 eV; O–O, 1.44 eV.

**Table 2:** Summary of experimental intercepts 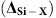 , 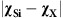 , ***χ***_X_ and Pauling's ***χ***_X_.*

**Target X**	**Intercept (eV)**	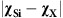	***χ***_**X**_	**Pauling's** ***χ***_**X**_
Si	–	–	1.8 (as reference)	1.8
Ge	0.00±0.07	∼0	∼1.8	1.8
Sn	0.00±0.06	∼0	∼1.8	1.8
Al	0.41±0.06	0.56±0.04	1.24±0.04	1.5
O	2.11±0.22	1.27±0.07	3.07±0.07	3.5
SiO_2_	0.26±0.08	0.45±0.07	2.25±0.07	–
SiNO	0.76±0.12	0.77±0.06	2.57±0.06	–

^*^in ref. 2.
