# Supplementary material for: Electronegativity determination of individual surface atoms by atomic force microscopy
Source: Nat Commun. 2017 Apr 26;8:15155. doi: 10.1038/ncomms15155 (PMC5414035; doi:10.1038/ncomms15155)
Supplement: Supplementary Information — Supplementary Figures, Supplementary Notes, Supplementary Methods and Supplementary References [file ncomms15155-s1.pdf]

Supplementary Methods 1

1.1 Germanium

For preparing Ge adatoms on the Si(111)-(7×7) surface, we used a filament-type source which wrapped a grain of Ge (Purity: 99.999 %). After preparing the clean Si(111)-(7×7) surface, we deposited Ge atoms on the sample at room temperature and then annealed at 600 °C typically for 5 minutes. A small amount of Ge caused the partial substitution of Si adatoms with Ge atoms. Identified Si and Ge at the corner adatom sites in faulted half (FH) unit cell are indicated by arrows in Supplementary Fig. 1a (or in the inset of Fig. 2a)[1]. In Supplementary Fig. 1b, we show the $\Delta f(z)$ curves on the Si and Ge adatoms as well as the BG $\Delta f(z)$ curve which includes only long-range contribution. The converted short-range (SR) force ($F_{SR}(z)$) and energy ($E_{SR}(z)$) curves are shown in Supplementary Figs. 1c and d, respectively. Note one has to approach the tip to at least the critical distance where $F_{SR}(z)$ reaches 0 nN so that $E_{SR}(z)$ can show the minimum potential energy. The maximum attractive forces of Si and Ge became 1.76 nN and 1.51 nN, while the minimum potential energies of them were 1.45 eV and 1.25 eV, respectively.

1.2 Tin

For preparing Sn adatoms on the Si(111)-(7×7) surface, we used a filament-type source which bound a fraction of pure Sn (Purity: 99.999%). After preparing the clean Si(111)-(7×7) surface, Sn atoms are deposited on the sample at room

temperature and then annealed at 600 °C typically for 5 minutes. A small amount of Sn caused the partial substitution of Si adatoms with Sn atoms as shown in Supplementary Fig. 2a (or in the inset of Fig. 2b). The brighter spots are Sn adatoms, which preferentially locate on the corner adatom site in FH unit cell. We performed site-specific spectroscopies on the Si and Sn adatoms at FH corner adatom cites indicated by arrows in Supplementary Fig. 2a. In Supplementary Fig. 2b, we show the curves acquired $\Delta f(z)$ on the Si and Sn adatoms as well as the BG $\Delta f(z)$ curve. The subtracted $\Delta f(z)$ curves were converted to $F_{\mathrm{SR}}(z)$ and $E_{\mathrm{SR}}(z)$ curves as shown in Supplementary Figs. 2c and d, respectively. The maximum attractive forces of Si and Sn became 0.78 nN and 0.56 nN, while the minimum potential energies of them were 0.91 eV and 0.64 eV, respectively.

1.3 Aluminum

For preparation of Al adatoms on the Si(111)-(7×7) surface, we used a crucible-type source which contained pure Al (Purity: 99.999%). After preparing the clean Si(111)-(7×7) surface, Al atoms are thermally evaporated and deposited on the sample at room temperature. Then, the sample was annealed at 600 °C typically for 5 minutes. As with the cases of Ge and Sn, a small amount of Al caused the partial substitution of Si adatoms with Al atoms. As shown in the inset of Supplementary Fig. 3a (or in the inset of Fig. 2c), the brighter spots are Al adatom substituted with Si adatoms. We carried out site-specific spectroscopies on the Si and Al atoms at FH corner adatom cites indicated by arrows in Supplementary Fig. 3a. We show the acquired $\Delta f(z)$ curves on the Si and Al

atoms as well as the BG $\Delta f(z)$ curve in Supplementary Fig. 3b, and converted $F_{SR}(z)$ and $E_{SR}(z)$ curves in Supplementary Figs. 3c and d, respectively. The maximum attractive forces of Si and Al became 0.93 nN and 0.89 nN, while the minimum potential energies of them were 0.63 eV and 0.73 eV, respectively.

1.4 Oxygen and SiO_2

In order to measure site-specific spectroscopies on the Si adatoms in SiO_2 and the O adatoms, we utilized initial (O-ins×2) and secondary (O-ad-ins×3) oxidation products, respectively. Here, notations of 'ins×*n*' (*n*=2 and 3) means *n* O atoms are inserted into *n* back-bonds of Si adatom, while 'ad' represents an O atom adsorbed on top of Si adatom. For preparing these products, we exposed the Si(111)-(7×7) surfaces to O_2 gas with the partial pressure of 2×10^{-9} Pa. Initial oxidation products start to appear from 0.01 L (1 L = 1 Langmuir) and kinetic analysis of initial and secondary oxidation products are previously examined by AFM in the exposure range from 0 L to 0.29 L[2]. Since O molecules preferably adsorb on the corner adatom site in FH unit cell and then form initial products of O-ins×2, subsequent secondary products of O-ad-ins×3 also tend to arise on the same site. Supplementary Fig. 4a represents the AFM image of the O-ins×2 and O-ad-ins×3 (simply denoted 'SiO_2' and 'O' in the main text, respectively) on the Si(111)-(7×7) surface. Apparently, the topographic height of latter one is larger than that of the former one. Supplementary Fig. 4b show the $\Delta f(z)$ curves acquired on the Si, O-ins×2, and O-ad-ins×3 at the FH corner site indicated by arrows in Supplementary Fig. 4a as well as the BG $\Delta f(z)$ curve. Converted

$F_{\mathrm{SR}}(z)$ and $E_{\mathrm{SR}}(z)$ curves of the relevant atoms are shown in Supplementary Figs. 4c and d, respectively. The maximum attractive forces of Si, O-ins×2, and O-ad-ins×3 became 0.86 nN, 0.92 nN, and 2.47 nN, while the minimum potential energies of them were 1.01 eV, 1.25 eV, and 3.26 eV, respectively.

Here, it is worth mentioning that whenever we scanned over O-ad-ins×3 with highly reactive tips, instability and subsequent tip-crash always arise just on the site. It seems that large chemical bonding energies causes the atom retraction from the tip apex to the surface O atom and vice versa. We also revealed by DFT calculations (not shown here) that the strong attractive force between a Si apex atom of a tip cluster and an O atom of the O-ad-ins×3 on the Si(111)-(7×7) surface cluster caused fatal destruction of the tip apex. Thus, such tip selection refrains us from obtaining the scatter plot of bond energies regarding with O at $E_{\mathrm{tip-Si}}$ > 1.5 eV (see Fig. 2d). On the other hand, this is not the case for O-ins×2, its scatter plot of bond energies can range from 0.5 eV to 2.5 eV as shown in Fig. 2e.

1.5 SiNO

For preparation of SiNO, which is introduced as NO-ins×2 here, we exposed the Si(111)-(7×7) surfaces to NO gas with the partial pressure of 2×10^{-9} Pa. Typically, after 0.02 L exposure of NO gas, bright spots appeared as shown in Supplementary Fig. 5a. As the case of O-ins×2, the NO molecule dissociatively adsorbs on the Si(111)-(7×7) surface, making the NO-ins×2 compound. Supplementary Fig. 5b shows the $\Delta f(z)$ curves acquired on the Si and NO-ins×2

indicated by arrows in Supplementary Fig. 5a as well as the BG $\Delta f(z)$ curve. Converted $F_{\mathrm{SR}}(z)$ and $E_{\mathrm{SR}}(z)$ curves of the relevant atoms are shown in Supplementary Figs. 5c and d, respectively. The maximum attractive forces of Si and NO-ins×2 became 0.97 nN and 1.90 nN, while the minimum potential energies of them were 0.67 eV and 1.32 eV, respectively.

Supplementary Methods 2

2.1 Cluster calculations for homogeneous bond energies

The formation energy of homonuclear bonds, which are introduced as homogeneous bond energies $E_{\mathrm{X-X}}$ in the main text, has been studied in a two-cluster geometry. The clusters were mirror images of each other with respect to a plane perpendicular to the z axis. The structure of the clusters for these calculations was derived from the Si_{15}(111)-T4 tip, with an apex modified accordingly to get the Ge, Sn, Al, SiO2, and SiNO terminations as shown in Supplementary Figs. 6a-f, respectively. The top and bottom Si layers of the clusters were passivated with H atoms. Only these hydrogen-passivated layers of Si atoms were kept in fixed position in order to define the distance between the tip and the surface. All other atomic positions were allowed to relax into equilibrium. The stopping criterion for the geometry optimization cycle was that the changes of total energy in subsequent iterations had to be below 10^{-6} eV.

2.2 Slab calculations

The (7×7) surface structure was represented by a slab consisting of two unreconstructed sub-surface Si bilayers and the three incomplete atomic layers which form Si(111)-(7×7) surface reconstruction (the stacking-fault layer, the restatom layer, and the adatom layer). The supercell was 3.6 nm high in the direction perpendicular to the surface, in order to provide enough space to accommodate also the tip (see the section of AFM simulations). The modified adatom structure (either by incorporation of O_2 or NO or substitution of Si by Al, Ge or Sn) was always placed into the FH unit cell adjacent to the corner hole. The bottom of the Si layer of the (7×7) substrate was passivated with H atoms. The parameters for obtaining equilibrium positions were the same as for the calculations with the mirror-geometry clusters. The local density of states (LDOS) of the optimized structures are shown in Supplementary Fig. 11. The projected electron density around the Fermi level is also represented in Supplementary Fig. 12.

2.3 Hartree potential

For the estimation of the work functions, computations of an unit cell of a modified Si(111)-(7×7) surface slab with Si, Ge, Al, O, SiO_2, or SiNO has been carried out as shown in Supplementary Fig. 13. Dipole compensation was switched on in this case in order to remove a spurious influence of electric field from neighboring slabs present because of periodic boundary conditions in the vertical direction. The basis defined by the 396 eV cutoff was used in these calculations.

2.4 Tip models

As shown in Supplementary Fig. 9, several different Si-atom terminated silicon clusters were used as tip models (bond energies on clean Si adatom at the FH corner site are also shown): (a) Si_{30}(001) dimer tip (1.26 eV), (b) Si_{31}(001) dimer tip (1.47 eV), (c) Si_{33}(001) tip (1.51 eV), (d) Si_{15}(111)-T4 tip (2.05 eV), (e) Si_{11}(111) dimer tip (2.08 eV), (f) Si_{10}(111)-H3 tip (2.16 eV). The subscripts in the tip nomenclature give the number of atoms, the Miller indices in the parentheses specify the orientation of the Si surface from which the base of the tip is derived. “H3” and “T4” denote, respectively, three-fold coordinated and four-fold coordinated site of the terminal Si atom, the “dimer” keyword says that the tip is terminated with a Si dimer.

2.5 AFM simulations

For the purpose of AFM simulations, computations with unit cells containing a modified Si(111)-(7×7) surface slab with a tip cluster have been carried out. A coarse approach of a tip towards the adatom site on the Si(111)-(7×7) surface was performed first for each combination of an adatom and tip model, in order to approximately localize the tip distance that corresponds to the total energy minimum. This coarse approach was done by steps of 25 pm with the 300 eV plane-wave cutoff. The energy minimum was then determined more precisely with a refined search in a limit range of tip distances with the distance step of 5 pm and the 396 eV plane-wave cutoff. The minimal value of total energy thus

found was then compared to the total energy for a tip placed far above the surface, about 600 pm, so as to obtain the bonding energy.

Supplementary Note 1: Equation for scatter plot of bond energies

Assuming that Pauling's equation regarding with polar covalent bonds holds at the potential minima in an interaction between foremost tip-termination and surface atoms, following relations are given;

$$E_{\mathrm{tip-X}} = \sqrt{E_{\mathrm{tip-tip}} E_{\mathrm{X-X}}} + \Delta_{\mathrm{tip-X}} \quad (3)$$

$$E_{\mathrm{tip-ref}} = \sqrt{E_{\mathrm{tip-tip}} E_{\mathrm{ref-ref}}} + \Delta_{\mathrm{tip-ref}} \quad (4)$$

where $E_{\mathrm{tip-X}}$ ($E_{\mathrm{tip-ref}}$) represents the heterogeneous bond energies between tip-termination and target X (reference "ref") atoms on a surface. $E_{\mathrm{tip-tip}}$, $E_{\mathrm{X\text{-}X}}$ and $E_{\mathrm{ref-ref}}$ indicate the homogeneous bond energies of the corresponding elements, respectively. $\Delta_{\mathrm{tip-X}}$ and $\Delta_{\mathrm{tip-ref}}$ are the ionic energies, which have been originally defined as the difference between the heterogeneous bond energies and the geometric means of homogeneous bond energies of constituting atoms according to Pauling. By substituting equation (4) for (3) so as to erase $E_{\mathrm{tip-tip}}$, the equations are unified into

$$E_{\mathrm{tip-X}} = \sqrt{\frac{E_{\mathrm{X-X}}}{E_{\mathrm{ref-ref}}}} \left(E_{\mathrm{tip-ref}} - \Delta_{\mathrm{tip-ref}} \right) + \Delta_{\mathrm{tip-X}} \ . \quad (5)$$

As described in the main text, given that assumed tip-termination and reference atoms are Si and their electronegativity values are almost identical, $\Delta_{\mathrm{tip-ref}}$ became almost zero. The equation (5) finally becomes equation (2).

Supplementary Note 2: Discussion of the electronegativity of Si tip

In principle, individual tips in the dataset of the scatter plot of bond energies could have different electronegativity values because the foremost tip atoms would have different coordination and hybridization with back-bonded Si atoms in the body part. However, the fitting process substantiates our assumption that electronegativity of Si tip (χ_{tip}) is almost the same as that of Si adatom on the Si(111)-(7×7) surface (χ_{Si}). Let us consider an equation (5) for an ith tip in the dataset of the scatter plot of bond energies, which is expressed as

$$E_{\text{tip}(i)-\text{X}} = \sqrt{\frac{E_{\text{X}-\text{X}}}{E_{\text{ref}-\text{ref}}}}\left(E_{\text{tip}(i)-\text{ref}} - \Delta_{\text{tip}(i)-\text{ref}}\right) + \Delta_{\text{tip}(i)-\text{X}} \,. \quad (6)$$

The electronegativity of the ith tip ($\chi_{\text{tip}(i)}$) can be described as

$$\chi_{\text{tip}(i)} \equiv \chi_{\text{Si}} + \delta\chi_i \quad \left(\delta\chi_i \le \left|\delta\chi_{\max}\right|\right) \quad (7)$$

where $\delta\chi_i$ is an electronegativity difference between the Si atom at the tip apex and the Si adatom on the Si(111)-(7×7) surface. Since tips with specific chemical reactivity and electronegativity values are not necessarily in one-to-one correspondence, $\delta\chi_i$ can have arbitrary value within the maximum range of $\left|\delta\chi_{\max}\right|$. Thus, when we choose the Si adatom on the Si(111)-(7×7) surface as reference atom and use equation (7), the individual ionic energies in equation (6) are written as

$$\Delta_{\text{tip}(i)-\text{Si}} = 1.3\left(\chi_{\text{tip}(i)} - \chi_{\text{Si}}\right)^2 = 1.3\left(\chi_{\text{Si}} - \chi_{\text{Si}} + \delta\chi_i\right)^2 = 1.3\delta\chi_i^{\,2} \quad (8)$$

$$\Delta_{\mathrm{tip}(i)-\mathrm{X}} = 1.3(\chi_{\mathrm{tip}(i)} - \chi_{\mathrm{X}})^2 = 1.3(\chi_{\mathrm{Si}} - \chi_{\mathrm{X}} + \delta\chi_i)^2 . \quad (9)$$

Then, the equation (6) is expressed as

$$E_{\mathrm{tip}(i)-\mathrm{X}} = \sqrt{\frac{E_{\mathrm{X-X}}}{E_{\mathrm{Si-Si}}}}\left(E_{\mathrm{tip}(i)-\mathrm{Si}} - 1.3\delta\chi_i^{\ 2}\right) + 1.3(\chi_{\mathrm{Si}} - \chi_{\mathrm{X}} + \delta\chi_i)^2 . \quad (10)$$

In order to see how the fluctuation of the tip electronegativity affects the scatter plot of bond energies, we simulated $\delta\chi_i$ by generating uniform random numbers and used equation (10) with the values of $\sqrt{\frac{E_{\mathrm{X-X}}}{E_{\mathrm{Si-Si}}}}$=0.56 and $\chi_{\mathrm{Si}} - \chi_{\mathrm{X}}$=0.56 for Al. The results are shown in Supplementary Fig. 7, where the values of $E_{\mathrm{tip}(i)-\mathrm{Si}}$ are referred to those used in Fig2c. If χ_{tip} for individual tips are the same as χ_{Si}, corresponding $\delta\chi_i$ become zero and the bond energies $E_{\mathrm{tip}(i)-\mathrm{Al}}$ are plotted exactly on the straight line for Al (Supplementary Fig. 7a). On the other hand, as $|\delta\chi_{\mathrm{max}}|$ increases from 0.05 to 0.5, a scattering from the straight line gradually becomes large as shown in Supplementary Fig. 7b to f. Although the experimental errors are not taken into consideration, the extent to which the plots are dispersed with $|\delta\chi_{\mathrm{max}}|$=0.05 or 0.1 (see Supplementary Fig. 7b and c) is similar to the experimental result in Fig. 2c. In order to see whether the case of $|\delta\chi_{\mathrm{max}}|$=0.05 or 0.1 is plausible, we investigated the degree of scattering using the square root of the residual sum of squares (σ), which is defined as

$$\sigma^2 = \frac{1}{n}\sum_{i=1}^{n}\left\{E_{\mathrm{tip}(i)-\mathrm{X}} - \left(\sqrt{\frac{E_{\mathrm{X-X}}}{E_{\mathrm{Si-Si}}}}E_{\mathrm{tip}(i)-\mathrm{Si}} + 1.3(\chi_{\mathrm{Si}} - \chi_{\mathrm{X}})^2\right)\right\}^2 . \quad (11)$$

Using equation (10), we can express equation (11) as

$$\sigma^2 = \frac{1}{n}\sum_{i=1}^{n}\left\{1.3\left(1-\sqrt{\frac{E_{\mathrm{X-X}}}{E_{\mathrm{Si-Si}}}}\right)\delta\chi_i^2 + 2.6\left(\chi_{\mathrm{Si}} - \chi_{\mathrm{X}}\right)\delta\chi\right\}^2 . \quad (12)$$

Then, we examined the probability distribution of σ by simulating 100 datasets of $\delta\chi_i$ with respect to $\left|\delta\chi_{\max}\right|$ =0.05, 0.1, and 0.02. As represented in Supplementary Fig. 8, the distributions of σ show a normal distribution. The mean values of σ for $\left|\delta\chi_{\max}\right|$=0.05 and 0.1 become 0.04 ± 0.01 and 0.09 ± 0.01, respectively. This means that we obtain these values with high probability if the electronegativity of Si tip fluctuates with $\left|\delta\chi_{\max}\right|$=0.05 and 0.1. On the other hand, in the cases of $\left|\delta\chi_{\max}\right| = 0.2$, the mean value of σ becomes 0.17 ± 0.02. Since we experimentally obtained σ =0.06 for Al (Fig. 2c) using equation (11), actual electronegativity of individual Si tips could fluctuate with $\left|\delta\chi_{\max}\right|$ ranging from 0.05 to 0.1. If the experimental tip electronegativity is deviated from χ_{Si} by more than 0.2, the degree of the scattering should be larger as indicated in Supplementary Fig. 7d to f. Moreover, σ that we would obtain should also be worse as shown in Supplementary Fig. 8. These analysis can rationalize our assumption that individual $\chi_{\mathrm{tip}(i)}$ are almost identical with χ_{Si}, namely,

$$\chi_{\mathrm{tip}} \approx \chi_{\mathrm{Si}} . \quad (13)$$

Supplementary Note 3: Theoretical scatter plot of bond energies

In order to corroborate further the existence of the intercept, we calculated the theoretical bond energies of Si and some impurities on the Si(111)-(7×7) surface

with various Si tips (shown in Supplementary Fig. 9). As shown in Supplementary Fig. 10, theoretical scatter plots of bond energies regarding with Ge, Al, and SiO_2 seem to match well with the experimental counterparts in Fig. 2. However, slopes and intercepts estimated only with the theoretical plots does not reproduce exactly the experimental values in Tables 1 and 2. The problem here is related to the absence of theoretical low-reactive Si-based tips, whose bond energies range from 0.5 eV to 1.0 eV for the Si adatom on the Si(111)-7x7 surface. Experimentally, it has been recognized that the chemical reactivity of Si tips can be changed in the previous studies[3, 4] as much as those in Fig. 2. In our calculations, although we tried to mimic low-reactive Si tips by adding dopants (such as Sb) close to the Si tip apex to change the electronic state, we found the models unstable under interaction with surface Si adatom. This points out the limits of our Si-tip models.

We think that the inherent instability of such doped tip models observed in our simulations is caused by their limited size (up to 48 Si atoms). In such small models, the impact of the doping atom is enormous being far from real doping concentration. On the other hand, a size extension of tip models makes not only these calculations computationally unbearable, but it also requires a more complex optimization of the atomic structure to avoid their structural instabilities under mechanical interaction due to chemical bond formation with surface adatoms. Such complex analysis is beyond the scope of this study.

The lack of the low-reactive Si tip models also prevents us to simulate the bond energies on O-adatom in O-ad-ins×3, where the reactive Si-tip models suffer

irreversible structural changes when they reach the maximum attractive force. The low-reactive tips could be reproduced if we take more tip sizes and realistic structures into account. However, as described above, precise calculations including the Si(111)-(7×7) slab and such tip models around the minimum energy are very expensive, and thus unfeasible. We need to count on development of computations in the future for tackling these problems.

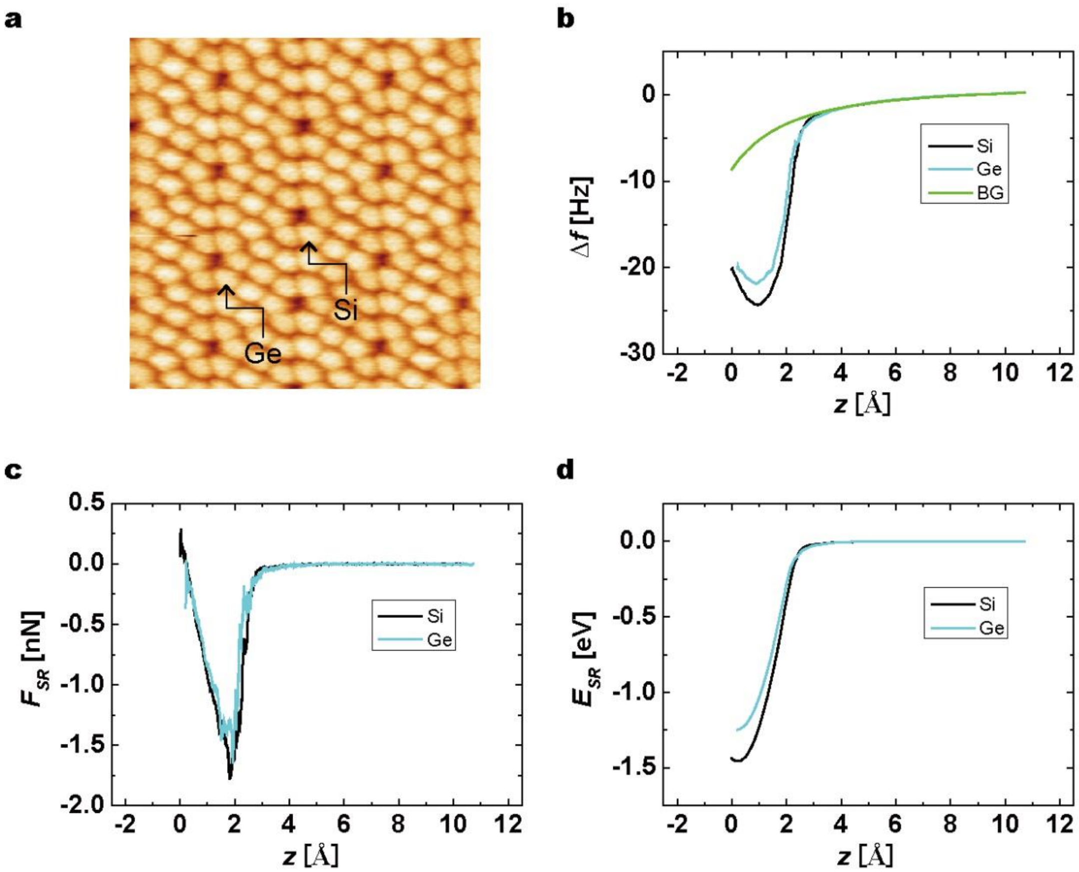

Supplementary Figure 1. Force and energy spectroscopies on a Ge adatom. **a** AFM topographic image of the Si and Ge adatoms located at FH corner sites on the Si(111)-(7×7) surface. Δf =-8.0 Hz. **b** $\Delta f(z)$ curves of Si, Ge and BG. Converted $F(z)$ **(c)** and $E(z)$ **(d)** curves of Si and Ge. The acquisition parameters are f_0=152.908 kHz, *A*=153 Å, *k*=28.2 N m^{-1}, V_S=-180 mV.

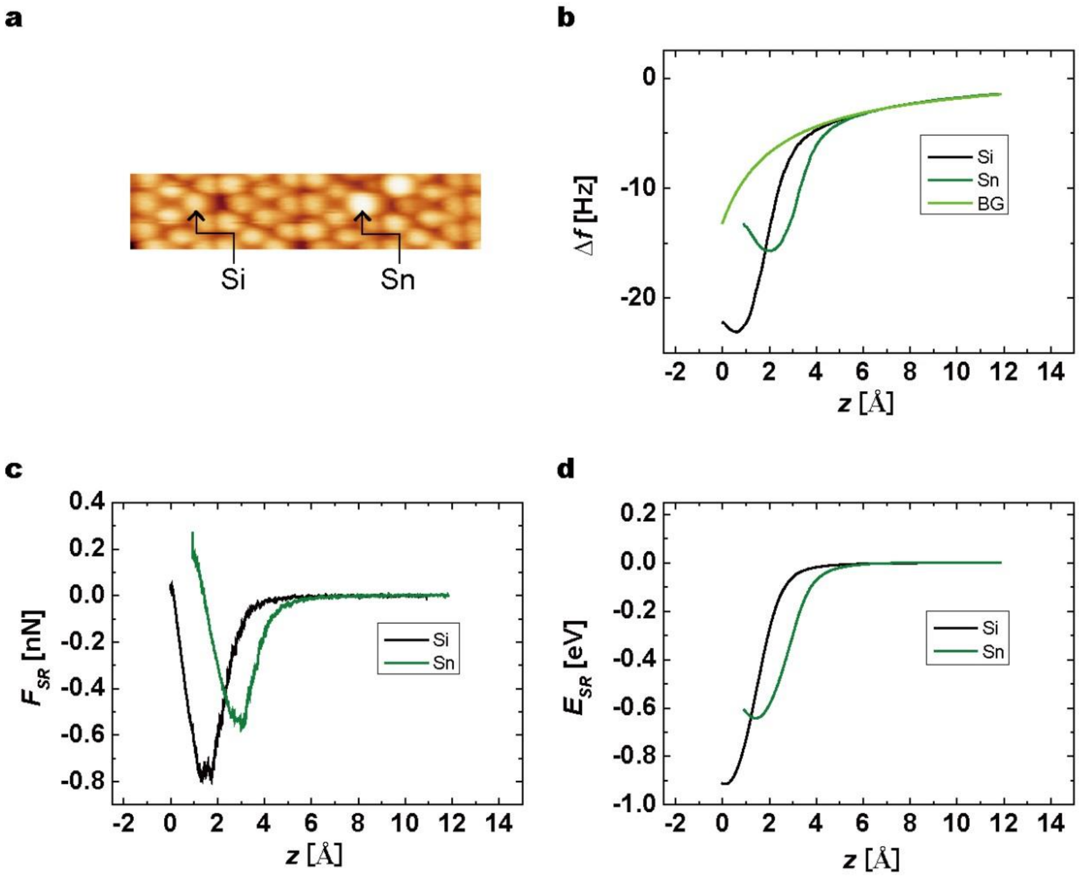

Supplementary Figure 2. Force and energy spectroscopies on a Sn adatom.

a AFM topographic image of the Si and Sn adatoms located at FH corner sites on the Si(111)-(7×7) surface. Δf =-9.7 Hz. **b** $\Delta f(z)$ curves of Si, Sn and BG. Converted $F(z)$ **(c)** and $E(z)$ **(d)** curves of Si and Sn. The acquisition parameters are f_0=156.243 kHz, A=135 Å, k=30.0 N m^{-1}, V_S=-230 mV.

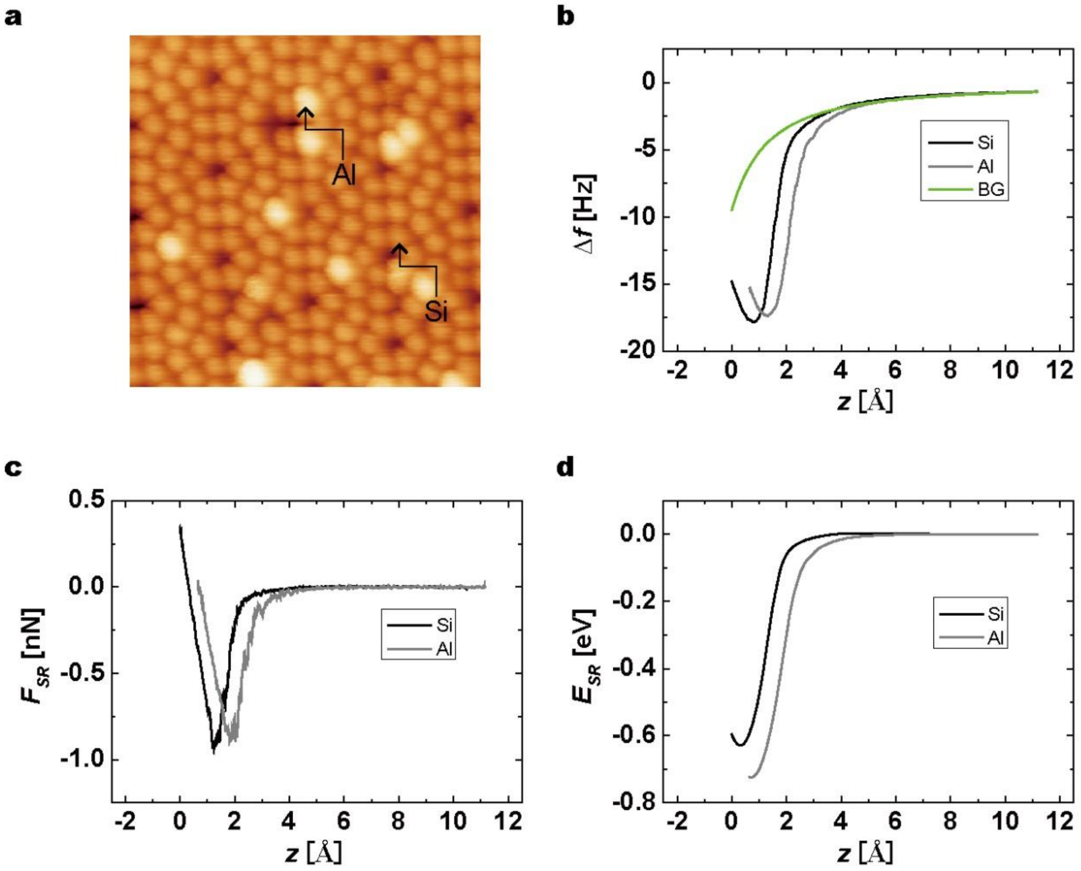

Supplementary Figure 3. Force and energy spectroscopies on an Al adatom. **a** AFM topographic image of the Si and Al adatoms located at FH corner sites on the Si(111)-(7×7) surface. Δf =-5.0 Hz. **b** $\Delta f(z)$ curves of Si, Al and BG. Converted $F(z)$ **(c)** and $E(z)$ **(d)** curves of Si and Al. The acquisition parameters are f_0 =158.563 kHz, A=133 Å, k=28.5 N m^{-1}, V_S=-82 mV.

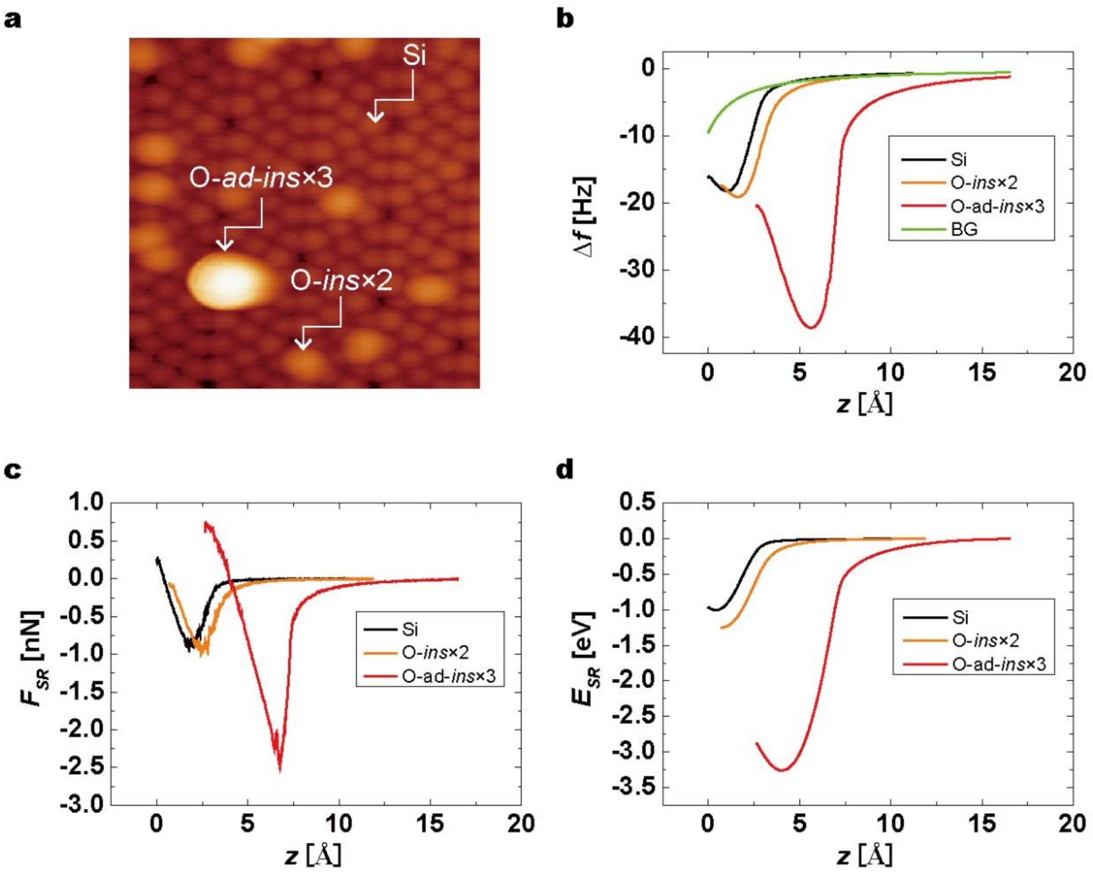

Supplementary Figure 4. Force and energy spectroscopies on O-ins×2 and O-ad-ins×3. a AFM topographic image of the Si adatom, O-ins×2, and O-ad-ins×3 located at FH corner sites on the Si(111)-(7×7) surface. Δf =-7.3 Hz. **b** $\Delta f(z)$ curves of Si, O-ins×2, O-ad-ins×3, and BG. Converted $F(z)$ **(c)** and $E(z)$ **(d)** curves of Si, O-ins×2, and O-ad-ins×3. The acquisition parameters are f_0 =152.913 kHz, A=146 Å, k=28.2 N m^{-1}, V_S=+40 mV.

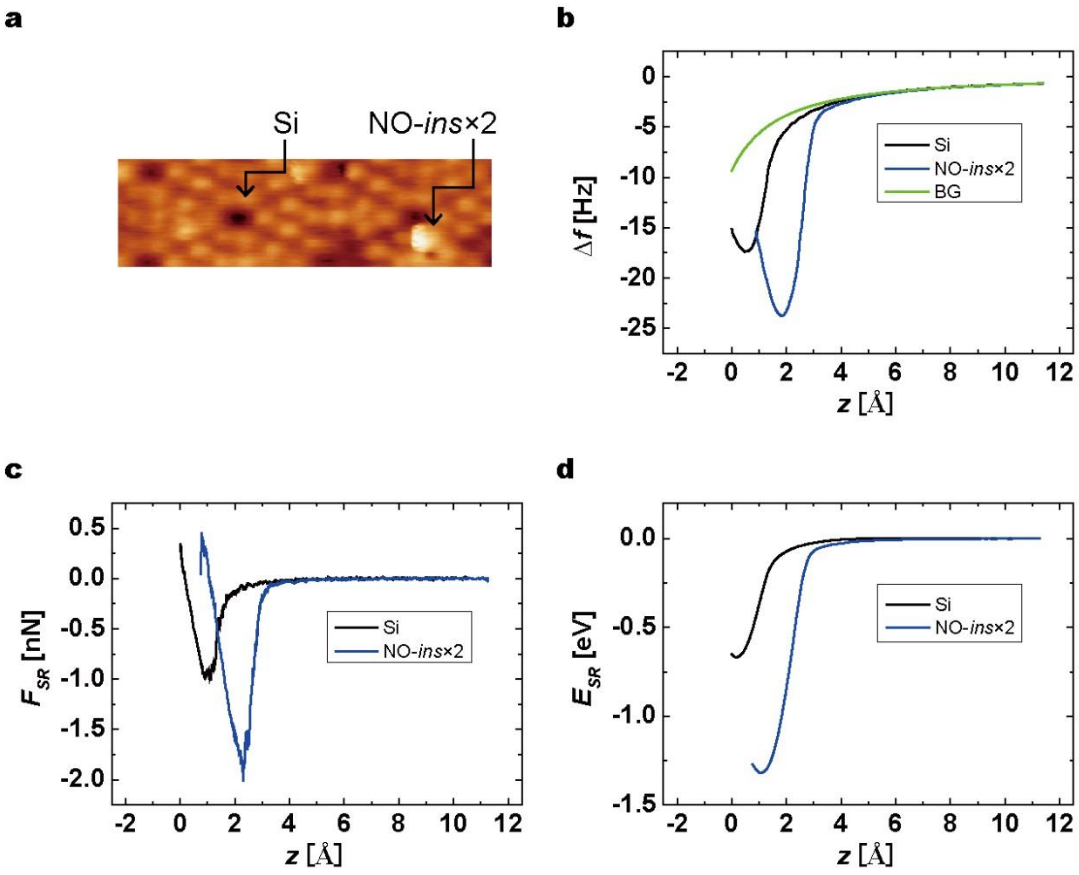

Supplementary Figure 5. Force and energy spectroscopies on NO-ins×2. a AFM topographic image of the Si and NO-ins×2 located at FH corner sites on the Si(111)-(7×7) surface. Δf =-5.1 Hz. **b** $\Delta f(z)$ curves of Si, NO-ins×2 and BG. Converted $F(z)$ **(c)** and $E(z)$ **(d)** curves of Si and NO-ins×2. The acquisition parameters are f_0 =155.818 kHz, *A*=150 Å, *k*=29.8 N m^{-1}, V_S=0 mV.

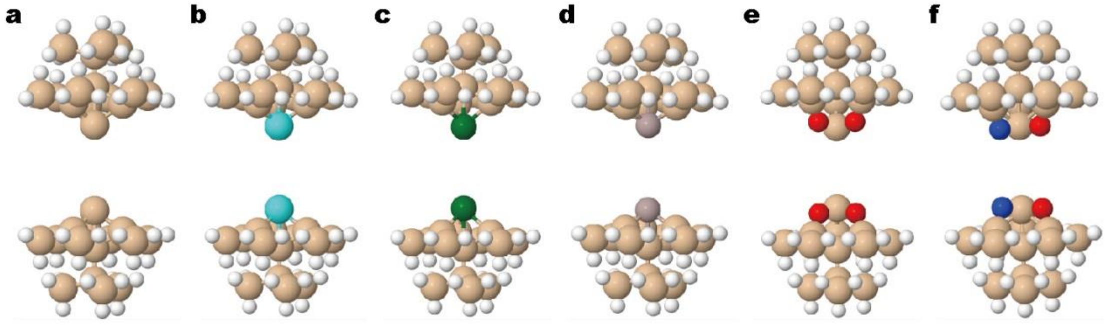

Supplementary Figure 6. Models for homogeneous bond energies. Mirror-symmetric cluster structures of Si (**a**), Ge (**b**), Sn (**c**), Al (**d**), SiO_2 (**e**), and (**f**) SiNO.

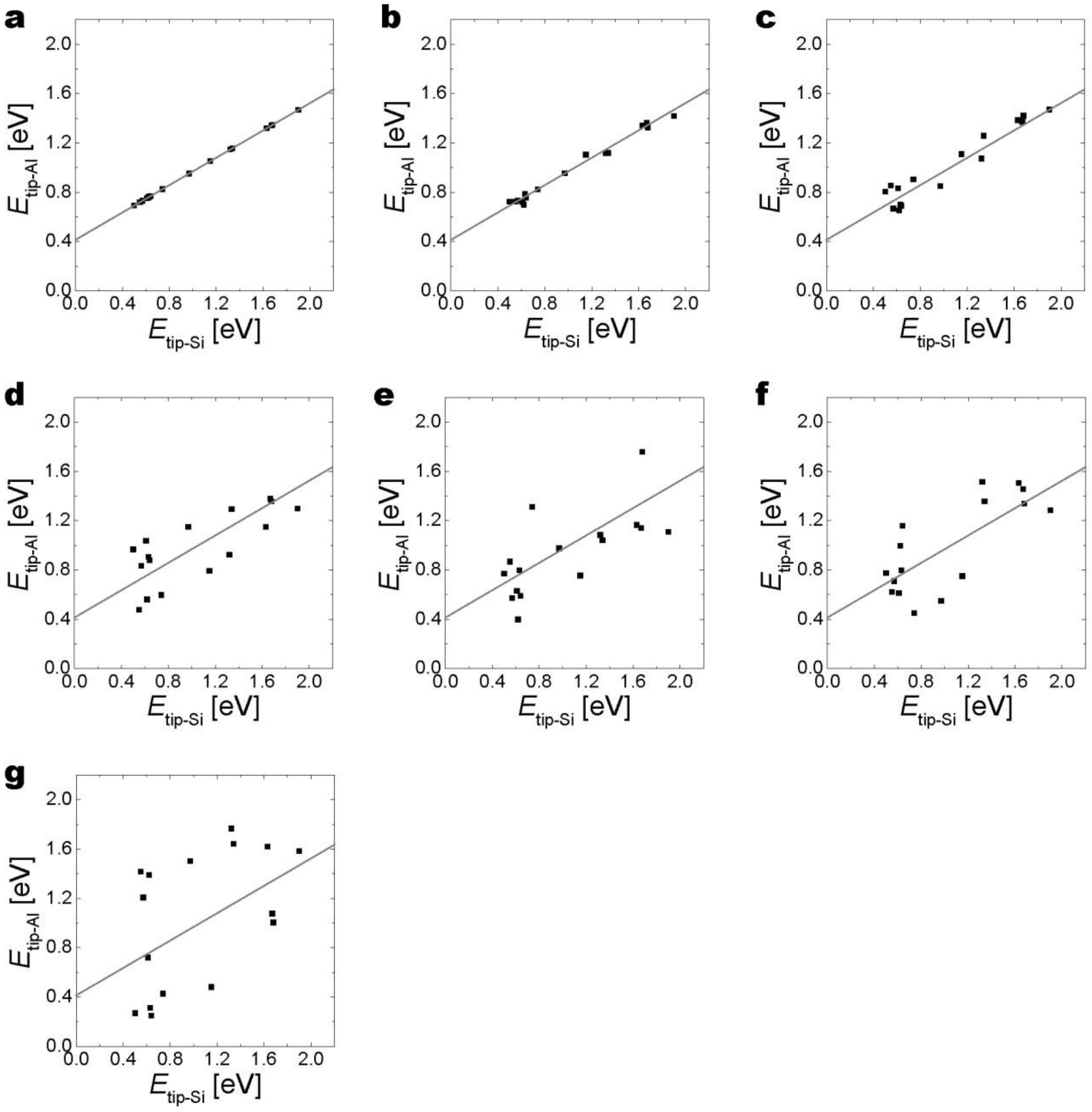

Supplementary Figure 7. Effect of the fluctuation of the tip electronegativity.

The scatter plots of bond energies for Al are simulated with parameters of $|\delta\chi_{max}|$ =0 (a), 0.05 (b), 0.1 (c), 0.2 (d), 0.3 (e), 0.4 (f), and 0.5 (g). Gray straight lines are the best-fitted line for Al shown in Fig. 2c.

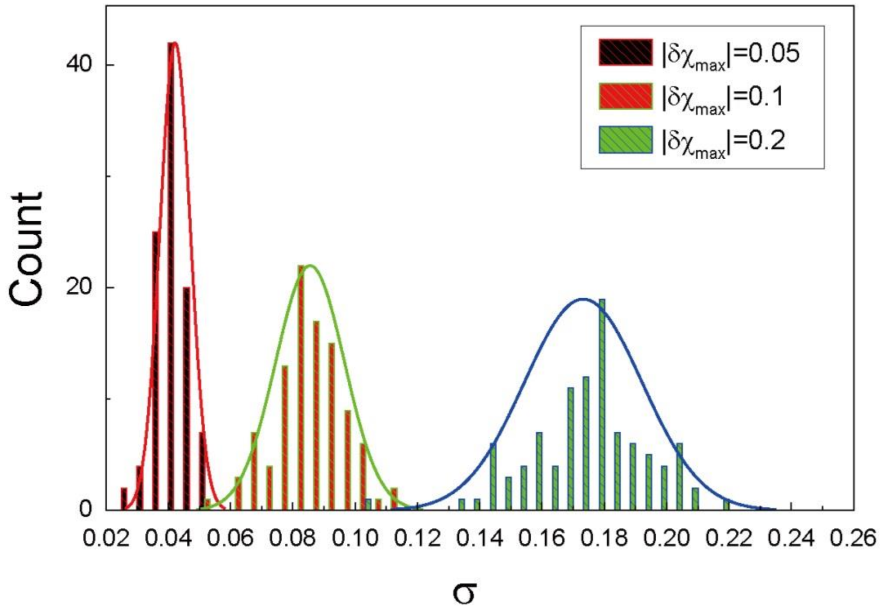

Supplementary Figure 8. Statistical analysis of σ . 100 datasets of $\delta\chi_i$ with respect to $\left|\delta\chi_{\max}\right|$=0.05, 0.1, and 0.02 are simulated, respectively

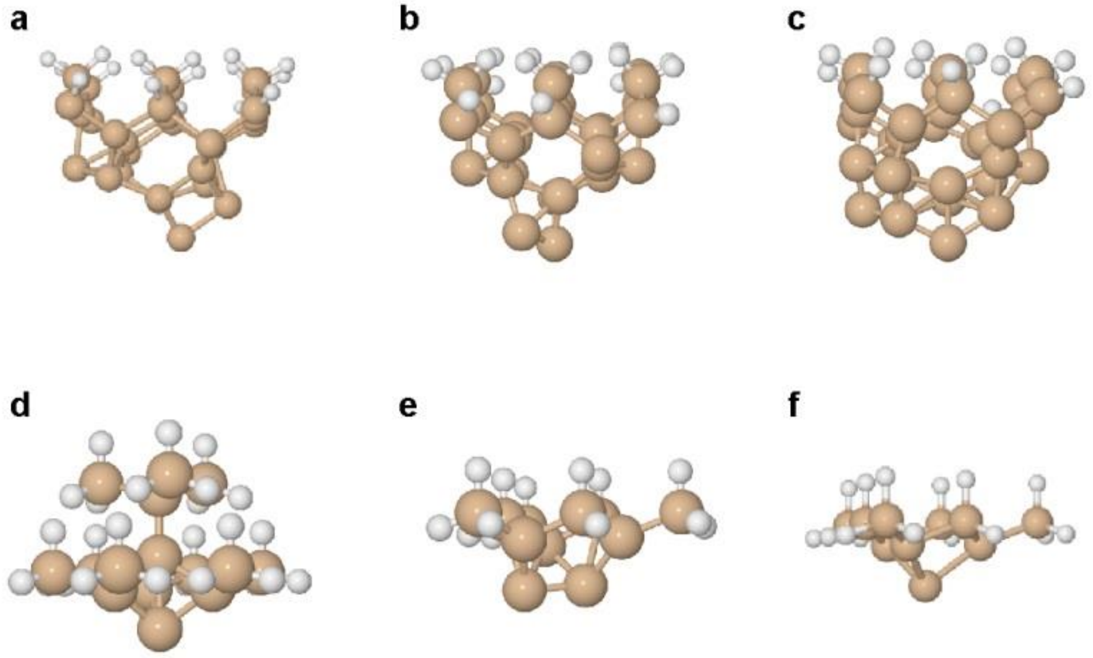

Supplementary Figure 9. Tip models used for the AFM simulations. Tip structures of Si_{30}(001) dimer (**a**), Si_{31}(001) dimer (**b**), Si_{33}(001) (**c**), Si_{15}(111)-T4 (**d**), Si_{11}(111) dimer (**e**), Si_{10}(111)-H3 (**f**).

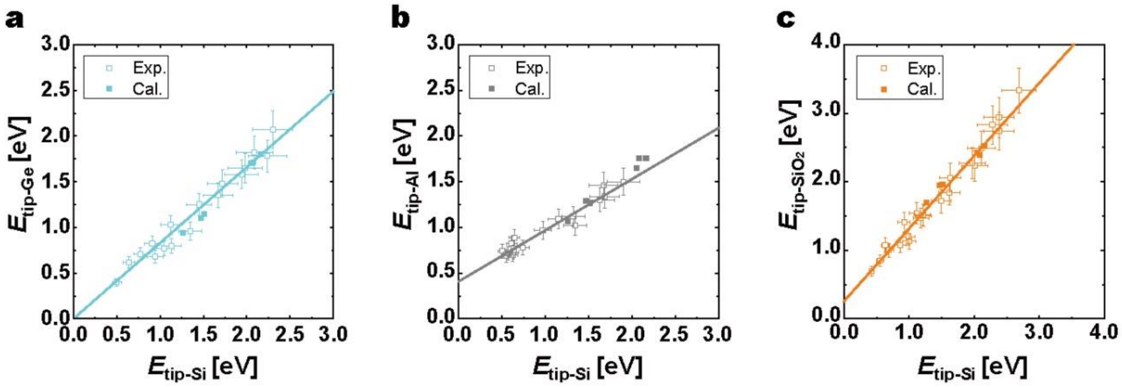

Supplementary Figure 10. Comparison of experimental and theoretical scatter plots of the bond energies. The scatter plots of Ge (**a**), Al (**b**), SiO_2(**c**). The theoretical scatter plots were obtained from total energy DFT calculations of the Si(111)-(7×7) slab containing single Ge, Al or SiO_2 with different Si tips (see Supplementary Fig. 9).

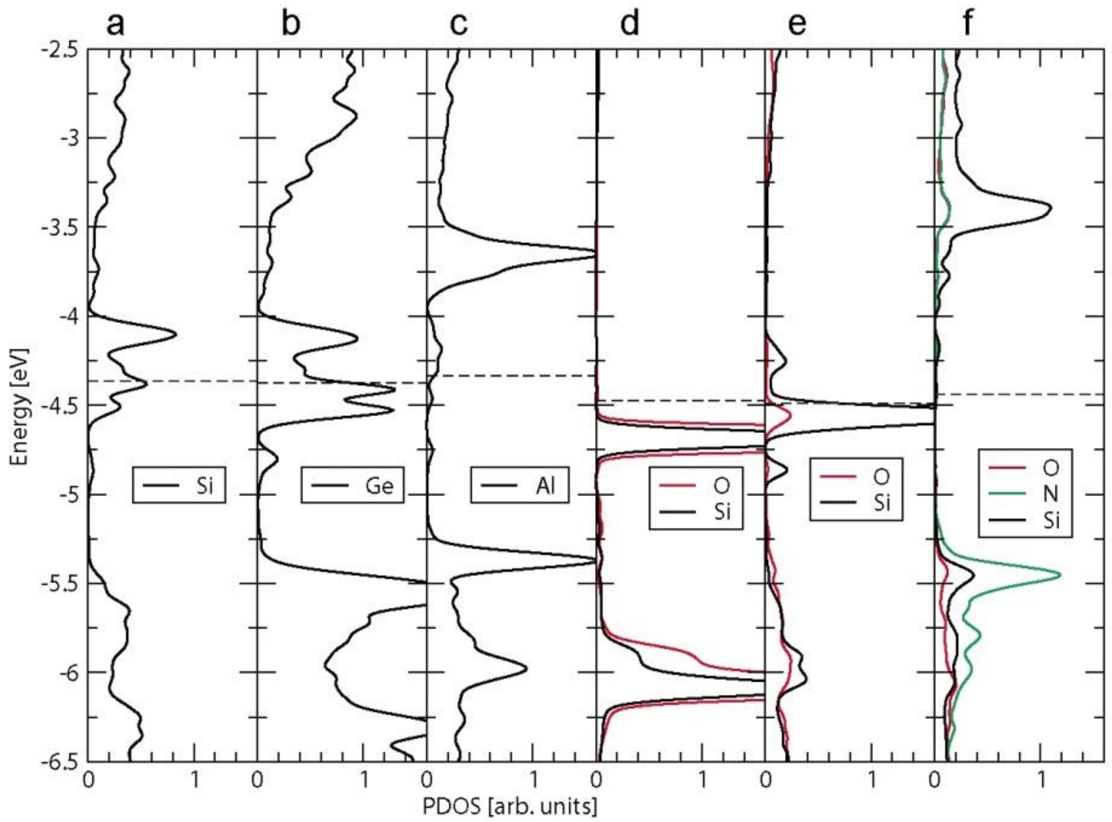

Supplementary Figure 11. LDOS of the impurities on the Si(111)-(7×7) surface. LDOS projected onto Si (**a**), Ge (**b**), Al (**c**), O (**d**) adatoms, SiO_2 (**e**), and SiNO (**f**) complexes.

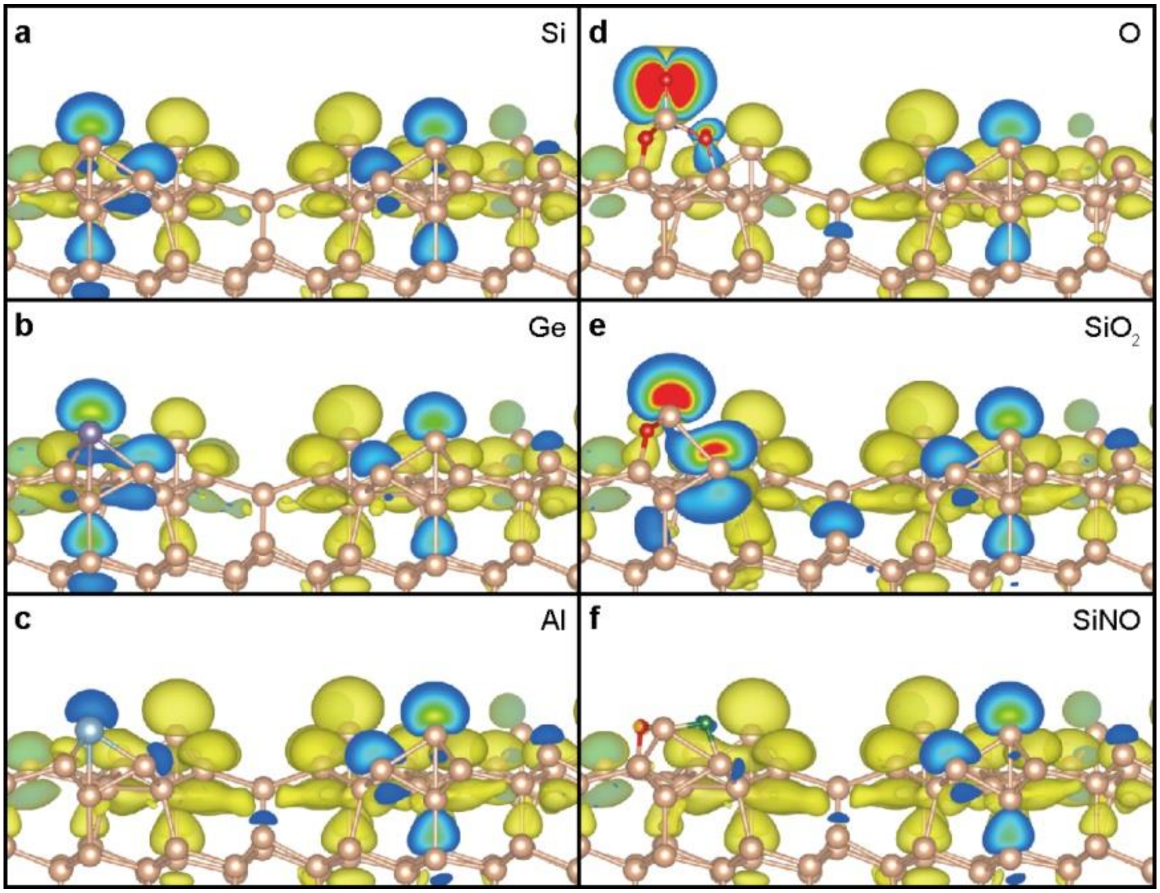

Supplementary Figure 12. Calculated electron density around the Fermi level. Electron density on Si (**a**), Ge (**b**), Al (**c**), O (**d**) adatoms, SiO_2 (**e**), and SiNO (**f**) complexes around the Fermi level in range of $E_F \pm 0.2$ eV, isosurface 0.005 eV Å^{-3}.

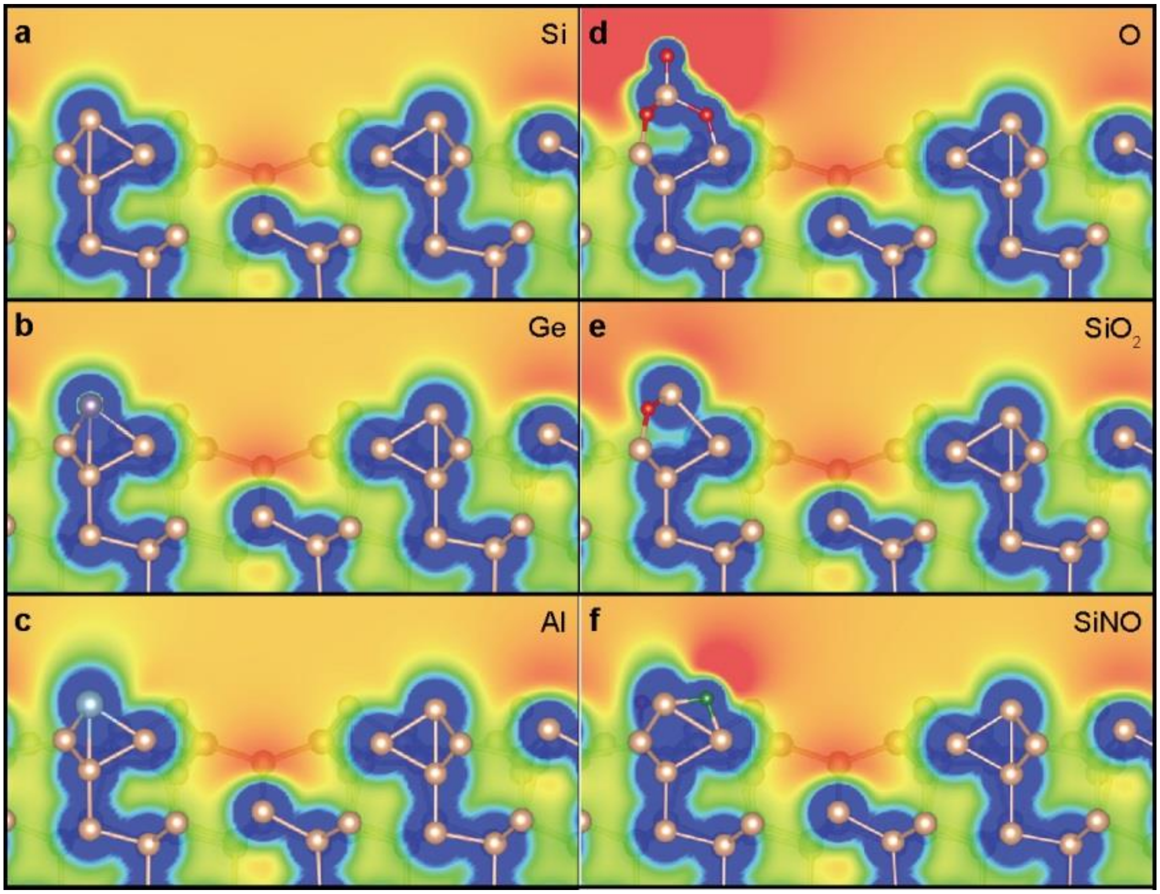

Supplementary Figure 13. Plotted 2D-plane of the Hartree potential. The Hartree potential projected onto Si (**a**), Ge (**b**), Al (**c**), O (**d**) adatoms, SiO_2 (**e**), and SiNO (**f**) complexes. Cut plane of the Hartree potential, color bar in range -1 (blue) to 5.5 (red) eV.

Supplementary References

1. Onoda, J., Niki K. & Sugimoto, Y. Identification of Si and Ge atoms by atomic force microscopy. *Phys. Rev. B* **92**, 155309 (2015).
2. Onoda, J., Ondráček, M., Yurtsever, A., Jelínek, P. & Sugimoto, Y. Initial and secondary oxidation products on the Si(111)-(7×7) surface identified by

atomic force microscopy and first principle calculations. *Appl. Phys. Lett.* **104**, 133107 (2014).

3. Yurtsever, A., Sugimoto, Y., Tanaka, H., Abe, M., Morita, S., Ondráček, M., Pou, P., Pérez, R., & Jelínek, P. Force mapping on a partially H-covered Si(111)-(7×7) surface: Influence of tip and surface reactivity. *Phys. Rev. B* **87**, 155403 (2013).
4. Sugimoto, Y. Yurtsever, A., Abe, M., Morita, S., Ondráček, M., Pou, P., Pérez, R., & Jelínek, P. Role of tip chemical reactivity on atom manipulation process in dynamic force microscopy. *ACS Nano* **7**, 7370-7376 (2013).
